# Evaluation of Quality Assessment Protocols for High Throughput Genome Resequencing Data

**DOI:** 10.3389/fgene.2017.00094

**Published:** 2017-07-07

**Authors:** Matteo Chiara, Giulio Pavesi

**Affiliations:** Dipartimento di Bioscienze, Università di MilanoMilan, Italy

**Keywords:** precision medicine, next-generation sequencing read quality, genome resequencing, whole exome sequencing, molecular diagnostics

## Abstract

Large-scale initiatives aiming to recover the complete sequence of thousands of human genomes are currently being undertaken worldwide, concurring to the generation of a comprehensive catalog of human genetic variation. The ultimate and most ambitious goal of human population scale genomics is the characterization of the so-called human “variome,” through the identification of causal mutations or haplotypes. Several research institutions worldwide currently use genotyping assays based on Next-Generation Sequencing (NGS) for diagnostics and clinical screenings, and the widespread application of such technologies promises major revolutions in medical science. Bioinformatic analysis of human resequencing data is one of the main factors limiting the effectiveness and general applicability of NGS for clinical studies. The requirement for multiple tools, to be combined in dedicated protocols in order to accommodate different types of data (gene panels, exomes, or whole genomes) and the high variability of the data makes difficult the establishment of a ultimate strategy of general use. While there already exist several studies comparing sensitivity and accuracy of bioinformatic pipelines for the identification of single nucleotide variants from resequencing data, little is known about the impact of quality assessment and reads pre-processing strategies. In this work we discuss major strengths and limitations of the various genome resequencing protocols are currently used in molecular diagnostics and for the discovery of novel disease-causing mutations. By taking advantage of publicly available data we devise and suggest a series of best practices for the pre-processing of the data that consistently improve the outcome of genotyping with minimal impacts on computational costs.

## Introduction

The steady reduction in sequencing costs associated with the advent of the new generation of ultra-high throughput sequencing platforms, collectively known as Next-Generation Sequencing (NGS) technologies, is one of the major drivers of the so called “genomic revolution.” Consequent to the development of these novel ultra efficient sequencing technologies [see (Goodwin et al., 2016) for a comprehensive review] the number of publicly available human genome and exome sequences is now in the hundreds of thousands, steadily increasing on a daily basis (Stephens et al., 2015). The characterization and fine scale annotation of the human *variome*, that is, the ensemble of genetic variants in the human population, is one of the most ambitious goals of massive human genome sequencing projects. The possibility to link genetic variants and haplotypes with the corresponding phenotypes and discover causal relationships or calculate risk factors is instrumental for the development of more informed approaches to medical science, such as precision medicine (Lu et al., 2014) where patients can be treated based on their genetic background, or predictive medicine (Kotze et al., 2015) where risks factors for various diseases can be calculated beforehand and suitable measures can be instituted in order to prevent the disease or decrease its severity. Numerous countries worldwide are currently undertaking or are planning to launch large-scale projects aiming to sequence an increasing proportion of their population: by example England (UK10K Consortium et al., 2015) and Saudi Arabia (Alkuraya, 2014) have both announced 100.000 individuals sequencing projects and researchers from the United States^1^ and China (Cyranoski, 2016) aim to sequence 1 million genomes in the next few years. In the meanwhile, various “pilot” (Gurdasani et al., 2015; Nagasaki et al., 2015; Sidore et al., 2015; The 1000 Genomes Project Consortium et al., 2015; Lek et al., 2016) projects, sequencing thousands of human genomes and exomes have been successfully undertaken which demonstrate the power of big data genomics for the identification of deleterious mutations and providing a substantial contribution to the understanding of the evolutionary processes that shape the genomes of modern human populations.

While the possibility to sequence an unprecedented number of individual genomes could serve as the basis for a new revolution in medical science and genetics, the need to handle, analyze and store huge amounts of data is posing major challenges to genomics and bioinformatics which at present remain largely unresolved. A possibly incomplete catalog of all known NGS sequencing platforms^2^ provide evidence for the presence of almost 2200 instruments worldwide, distributed in 1027 sequencing facilities across 62 countries. A conservative estimate of sequencing capacity based on the manufacturer specifications of the various instruments suggests that, if used at full scale, NGS platforms could generate in the excess of 35 petabases of sequencing data per year. In a recent paper Stephens et al. (2015) suggest that, at the current rate, worldwide sequencing capacity could possibly reach zettabases of sequencing in the next 10 years, corresponding a number of complete human genomic sequences ranging from 100 million to 2 billion.

Bioinformatic analysis is currently one of the major bottlenecks in the processing of human resequencing data (Alyass et al., 2015). The need to integrate multiple tools into dedicated and sometimes complex analysis procedures requires a substantial amount of manual work and represents a major hindrance that limits the speed and general applicability of genotyping strategies. While best practices, procedures, and guidelines, defining the basic principles for the analysis and the annotation of the data have been introduced (Richards et al., 2015) and a large collection of bioinformatic tools for the identification of simple nucleotide variants (SNV) and/or small indels from NGS resequencing data is currently available (Pabinger et al., 2014), there exists no golden standard approach, and comparative studies evaluating different genotyping pipelines reached contrasting conclusions (Cornish and Guda, 2015; Hwang et al., 2015; Zhang et al., 2015). Consensus call-set based approaches, integrating predictions from multiple tools, can improve the accuracy and sensitivity of genotyping strategies (Bao et al., 2014). They, however, require additional computational costs, which might not always be justified by improvements in accuracy. Also, notwithstanding the high standardization of laboratory protocols and kits used in their production, the variability of NGS sequencing data remains high, and systematic biases resulting in the so called “batch effects” (Taub et al., 2010) can limit the extent of any bioinformatic approach, preventing the development of a conclusive strategy.

While several studies that compare the performances of various tools and pipelines are currently available [see (Pabinger et al., 2014), for a comprehensive review], at the time being we are not aware of dedicated studies evaluating the effects of quality assessment and reads pre-processing strategies in genotyping studies. Fine scale optimization of such procedures can on one hand improve the accuracy of the results, and on the other reduce significantly the computational requirements. This step is therefore essential as the starting point in the development of highly scalable workflows for the analysis of human resequencing data. In this article we discuss major strength and limitations of the different types of resequencing protocols that are currently used in molecular diagnostics and population scale genomics, and, by taking advantage of publicly available data, we devise guidelines and best practices for the pre-processing of the sequences.

## Resequencing Strategies, Applications and Limitations

The ability to perform population scale studies of large genomes is highly interlinked with the advent of modern ultra-high throughput DNA sequencing technologies and the substantial reduction in sequencing costs. At present, Illumina accounts for the largest share of the sequencing market. The recent release of the ultra-high throughput Illumina X Ten sequencing system^3^, which permits the sequencing of 1000s of human genomes per year for less than 1000 USD per genome, represents a major breakthrough in this field, and at the time being all the most ambitious population scale human genome resequencing projects are based on this technology. One of the major constraints of the second generation of ultra-high throughput sequencing technologies is the reduced size of the reads (a few hundreds bps), that poses limits to the possibility of reconstructing accurately long haplotypes and resolve repetitive and complex regions, which represent approximately 60% of the human genome (De Koning et al., 2011). Such limitations are being superseded by the development of a third generation of NGS sequencing platforms such as the PacBio (Eid et al., 2009) and Oxford Nanopore (Clarke et al., 2009) sequencing systems, that can produce sequences of stretches of DNA ranging from a few to hundreds of kilobases in size. Such long reads can span complex or repetitive regions with a single continuous read, thus eliminating ambiguity in the positions or size of genomic elements. A recent resequencing of the human GRCh37 reference genome based on long-read sequencing technologies (Chaisson et al., 2015) recovered more than 1 Mb of novel sequence and identified more than 26,000 relatively long (≥50 bp) indels, providing one of the most comprehensive genome reference sequences available. Apart from simply improving reference genomes, long reads are more effective than short reads in the identification of clinically relevant structural variations and in the reconstruction of long haplotypes (Ammar et al., 2015).

The comparatively higher error rate (15% on the average) and the increased cost with respect to short read technologies are considerable disadvantages that limit the application of long-read sequencing in current large-scale genome resequencing studies. However, the continuous improvements in sequencing chemistries and base calling algorithms, and the development of novel sequencing platforms with reduced operating costs such as the PacBio Sequel or the Oxford Nanopore Promethion might open the possibility of applying long-read sequencing technologies to population scale resequencing projects in the near future. In this respect, the recent development of sequencing strategies for the generation of synthetic long reads relying on existing Illumina platforms, with error profiles and throughput similar to those of current Illumina devices, might represent a valid alternative to long-read sequencing technologies. Two different systems for generating synthetic long-reads are currently available: the Illumina synthetic long-read sequencing platform (McCoy et al., 2014) (formerly known as Moleculo) and the 10X Genomics emulsion-based system^4^. Both platforms rely on a similar strategy, where a specialized library preparation method based on extreme dilution and DNA barcoding is applied to a size selected DNA library in order to mimic single molecule sequencing. Input DNA is first sheared into kilobase-long fragments, which are then randomly distributed across a small number of containers. The contents of each container are then sheared further into shorter fragments and are assigned a unique barcode before being pooled together for sequencing. After sequencing, reads are demultiplexed using the barcodes. Each container may be assembled separately with a short-read assembler, which produces multiple kilobase-long sequences in each well; this approach is referred to as subassembly. Alternatively, the contents of each container may be sequenced at a relatively low coverage and resulting reads might be used to assist in tasks such as genome phasing and scaffolding. The main difference between the two approaches consists in that while the Illumina system is aimed at the precise reconstruction of each long DNA fragment and is designed for genome assembly, the 10X strategy does not attempt gapless, end-to-end coverage of single DNA fragments, and is generally used for haplotyping and scaffolding. Considerations on sequencing costs, however, suggest that, for the time being, complete genome assembly of complex genomes based on synthetic long-reads technologies remains unfeasible, even if using the most advanced sequencing machines.

Hybrid approaches based on the combination of long or synthetic long and short reads have proven themselves to be highly effective in the generation of high-quality assemblies of large and complex genomes at a relatively low cost, resulting in the detection of complex structural rearrangements and in the accurate reconstruction of haplotypes (Mostovoy et al., 2016; Collins et al., 2017; Weisenfeld et al., 2017). With the ongoing steady reduction in sequencing costs it is not unfeasible to imagine that strategies of this kind will end up to be applied also to large-scale sequencing studies, resulting in a more accurate reconstruction of individual genomes and extending our understanding of the human variome.

Large-scale genome resequencing studies are nowadays usually performed by two alternative approaches: Whole Genome Shotgun sequencing (WGS), that is, the sequencing of complete genomes, or targeted resequencing, where high-throughput sequencing is applied to a predefined subset of genomic loci, usually selected on the base of their annotation (i.e., exons) or their association with pathological conditions. WGS clearly offers a more comprehensive, virtually complete, catalog of the genetic variation of an individual and is not limited by prior knowledge of the sequence, permitting the reconstruction of complex genomic rearrangements and large insertions. On the other hand, targeted resequencing, by limiting the size of the genomic material used, makes possible the sequencing of several samples within a sequencing run, increasing both the breadth and the depth of a genomic study on the selected loci. Another considerable advantage of targeted resequencing is that newly identified variants are more easy to interpret and characterize, since target regions usually correspond to functionally annotated genomic loci. Considering that the majority of known disease causing mutations is found in protein coding genes, the wealth of data produced by WGS approaches might result excessive and sometimes even misleading for clinical and diagnostic applications. Recent studies (Belkadi et al., 2015), however, suggest that WGS resequencing data are in general of better quality than the targeted resequencing counterpart, resulting in a slightly improved power in the detection of novel mutations even within targeted regions. Moreover, targeted resequencing can interrogate only predefined regions of the genome, and is therefore clearly ineffectual in the detection of large chromosomal rearrangements and large structural variants.

### Whole Exome Sequencing

The deep sequencing of all the exons of a genome, known as Whole Exome Sequencing (WES) (Ng et al., 2009), is probably the most popular and widely used targeted resequencing approach. As the name suggests, it is based on exome capture, that is, the construction of DNA libraries enriched for the exonic fraction of the genome. DNA samples are randomly fragmented and oligonucleotide probes (baits) are used to capture the target regions by DNA hybridization. The resulting DNA sample is then subjected to library construction and sequencing. Whole-exome methods generally capture from 35 to 100 megabases of DNA target regions, depending on the reference annotation system used in the design of the probes and on the inclusion of 3′ or 5′ untranslated regions (UTRs) in the experimental design. Agilent, Nimblegen, Illumina are the main suppliers of exome-enrichment kits for exome capture. The most relevant differences between these technologies are in the choice of target regions, in bait lengths and density, in the molecules used for capture, and in the genome fragmentation method. At present NimbleGen offers the largest target region set, covering 96 Mb (64 Mb coding + 32 Mb UTR), compared to 75 Mb (50 Mb coding + 25 Mb UTR) of Agilent and 62 Mb (42 Mb coding + 20 Mb UTR) of Illumina. However, given the continuous improvements in sequencing throughput of NGS sequencing technologies, the range of genomic regions targeted by exome capture kits is constantly expanding, resulting in the inclusion in target regions of promoter regions and intron-exon junctions (Samuels et al., 2013).

#### Whole Exome Sequencing in Clinical Studies

Since the majority of known disease-causing mutations are found in protein coding genes, WES is becoming an increasingly attractive alternative to WGS for clinical applications. As an additional advantage, genotyping assays performed by exome sequencing have a narrow breadth if compared with WGS approaches, and thus require less computational resources for the analysis and the storage of the data. Moreover, novel genomic variants discovered by exome sequencing are restricted to functionally annotated genomic regions, thus enabling a rapid inference of potential functional effects. Finally, notwithstanding the additional costs required for the capture kits, exome sequencing remains more economic than WGS, making possible the sequencing of a higher number of samples with an increased depth of coverage. For all these reasons, despite the increasing number of completely sequenced human genomes, WES sequencing remains today the preferential strategy for large-scale sequencing studies and for clinical applications of genome sequencing, and indeed the majority of available human resequencing data is in the form of exomes (Lek et al., 2016). This is also reflected in primary repositories of human genetic variation data, as in the latest release of the dbSNP database (Sherry et al., 2001), where the number of SNPs falling into protein coding genes surpasses by far the number of those found in intergenic regions (dbSNP build 141).

The relatively heterogeneous profile of read coverage over target regions is one of the major bottlenecks that reduce the sensitivity and applicability of exome capture assays. Experimental biases resulting in the so called “batch effects” are generally introduced both during exome capture and in the library preparation steps (Chilamakuri et al., 2014; Shigemizu et al., 2015). Such biases are specific and intrinsic to the different capture kits and library preparation protocols, and therefore limit the possibility of comparing WES experiments performed by means of different capture kits or by different sequencing providers. Also, in a typical exome sequencing study, approximately 40–60% of the reads derive from genomic regions outside of the designed targets, resulting in a substantial reduction of the theoretical coverage. Exome capture efficiency is highly variable and influenced by multiple factors related both to the design of the capture kit (length of the probes, probes density, probes design) and to experimental conditions affecting the efficiency of DNA fragmentation and PCR amplification of the DNA library (García-García et al., 2016).

Another relevant bias introduced by the capture hybridization step in WES sequencing consists in the preferential capture of reference sequence alleles, which hinders the detection of alternate alleles at heterozygous polymorphic sites by shifting the allele distribution (Guo et al., 2013). Highly polymorphic and heterozygous genomic regions are thus captured at lower efficiency than highly conserved genomic intervals, resulting again in a systematic bias in the coverage profile. Moreover, all library preparation protocols for exome sequencing require PCR amplification, which tends to lower coverage in GC rich regions due to annealing during amplification (Aird et al., 2011). Fluctuations in the coverage profile have a deep impact on the sensitivity of WES, and in particular in the detection of heterozygous variants (Belkadi et al., 2015). It has been estimated that 15X mapped read depth of WGS samples would be sufficient to detect almost all homozygous SNPs and 33X for almost all heterozygous SNPs (Bentley et al., 2008). Depending on the capture kit, it has been shown that WES required 80X mean on-target depth to reach the common threshold of 10X per-site depth in 90% or more of all targeted regions (Clark et al., 2011), which represents the minimal requirement for clinical applications of the WES technology.

### Gene Panels

Gene panels are another popular form of targeted resequencing which is often used in large diagnostic screenings. This approach leverages on prior knowledge about the association of a set of genomic loci with phenotypic traits of interest (typically a disease) in order to perform highly focused sequencing of a very specific portion of the genome (Katsanis and Katsanis, 2013). Loci of interest, ranging to a few kilobases to several megabases in size, are usually enriched either by DNA hybridization capture or by targeted amplification (amplicon sequencing), and then sequenced with high-throughput sequencing platforms. Enrichment systems based on PCR amplification require a very limited quantity of DNA for the construction of the sequencing library, thus enabling the analysis of relatively tiny tissue samples which are common in medical applications. The capture of target regions is highly specific and does not suffer from off-target DNA contamination, offering a substantially higher coverage. Differential PCR amplification of the target regions, however, can introduce relevant biases, resulting in a highly heterogeneous coverage profile (Samorodnitsky et al., 2015). Depending on PCR primers design and of DNA fragmentation accuracy, target regions might end up to be covered only by reads obtained from a single DNA strand, resulting in a considerably higher error rate due to the fact that second generation NGS technologies are affected by systematic context-specific sequencing errors (Schirmer et al., 2015).

Capture systems based on DNA hybridization show better coverage uniformity, higher sensitivity and better accuracy than amplicon-based methods. Moreover, since the size of target regions is not strictly limited by PCR primers, capture by hybridization can recover also relatively small (depending on the size of the baits) structural variants, which are systematically missed by amplicon sequencing techniques. The amount of off-target capture is comparable to WES, accounting for about 40–50% of the reads: this proportion can, however, vary greatly according to the design of the array, since low complexity and micro-satellite regions, which are often found in intronic sequences, can sensibly reduce the specificity of the capture. Capture hybridization systems are more expensive and require a considerably larger amount of DNA for library construction if compared with equivalent amplicon-based strategies, making them a less attractive option for high-throughput genotyping of large cohorts of samples.

#### Gene Panels in Clinical Studies

Gene panels are particularly suited for diagnostic screenings, since they provide a consistent reduction in costs and turnaround times and offer the possibility to customize the design of the panel in order to include complete genes or specific intronic sequences.

High-throughput sequencing of a limited number of carefully selected loci enables the characterization of wide cohorts of patients, virtually querying the presence of all known causal mutations and therefore providing an invaluable tool for diagnostics. Large-scale screenings based on carefully designed gene panels show a diagnostic power comparable, or even superior to that of WES, as the reduction in sequencing costs permits the sequencing of larger cohorts of patients (Saudi Mendeliome Group, 2015). This strategy, however, requires a substantial knowledge of the molecular basis of the condition/disease under study, and is not clearly applicable to the discovery of novel disease-causing mutations affecting genes not previously associated with the condition of interest.

Gene panels sequencing typically result in a very large of coverage of the target regions, exceeding 1000X in most cases. This coverage depth surpasses by far the minimum requirements for genotyping applications, and enables the reliable detection of somatic variants that might be present in a minority of the cellular population. The possibility to detect somatic variants in heterogeneous cellular populations is a very powerful tool for cancer genomics. Since carcinogenesis is an evolutionary process driven by natural selection, tumors of all types consist of cellular populations that are highly diverse at the genetic, epigenetic, and phenotypic levels. Tumor heterogeneity is a major cause of therapy failure and disease resistance, and is a subject of the utmost biological and clinical relevance. Ultra-high coverage targeted resequencing of panels of known tumor related genes thus enables the characterization of cancer cell populations and the detection of somatic cancer mutations, including those possibly linked with drug resistance (Gerlinger et al., 2012; Kim et al., 2015; Au et al., 2016; De Leng et al., 2016), a process that can be instrumental for the correct formulation of personalized anti-tumoral therapies. Repeated sequencing over time permits to monitor the evolution of the tumoral population in response to therapies, both for the evaluation of the efficacy of the therapy, by studying the prevalence of “founder mutations,” and for the identification of possible new resistance inducing variants. Therapies can be thus adapted accordingly, maximizing their efficacy.

### Whole Genome Sequencing

Whole genome shotgun sequencing (WGS) is rapidly becoming the method of choice for the study of human genetic variation at population scale level. Indeed, recent studies (Belkadi et al., 2015; Meienberg et al., 2016) suggest that, beside the capacity to interrogate a substantially larger fraction of the genome, WGS can offer major advantages and data of superior quality with respect to targeted resequencing approaches.

Whole genome shotgun-based strategies are not based on prior knowledge of the reference genome and can (in principle) address any type of complex genomic structural variant, including inversions, large insertions and deletions. The relevance of structural events of this type has been largely underestimated, since it is now clear that they contribute more than SNVs to the variability of individual genomes (Huddleston et al., 2016), where they can constitute up to 75% of the individual specific genomic material.

Whole genome shotgun sequencing libraries require a simpler and more streamlined preparation, where the most recent protocols do not require PCR amplification resulting in a substantially more homogeneous coverage profile (Meienberg et al., 2016). Target regions capture, that as previously discussed can introduce significant amounts of technical variability, is in turn not required by WGS. As a consequence, WGS data show consistently superior coverage uniformity with respect to WES, and a substantially lower average read depth is required to achieve the same breadth of coverage (Belkadi et al., 2015). These facts permit a more consistent identification of heterozygous mutations, and a more reliable discovery of copy number variants (Belkadi et al., 2015; Meienberg et al., 2016). More importantly, WGS does not suffer from reference bias capture, resulting in a more accurate calling of heterozygous variants. Finally, while all the commercially available exome capture kits are prone to systematic biases that are in large part platform specific (Chilamakuri et al., 2014; Shigemizu et al., 2015; García-García et al., 2016) and limit the possibility to compare data across different systems and kits, WGS data are to some extent more reproducible and comparable, facilitating the comparison of data produced by different sequencing facilities at different times.

#### Whole Genome Sequencing in Clinical Studies

The major factors limiting the adoption of WGS technologies in clinical practice are not only related to the increase in costs with respect to targeted resequencing, but also to the computational resources required for the bioinformatic analysis and interpretation of the data. A typical human genome contains approximately 4 million SNV or small indels (Eberle et al., 2016), the vast majority of which is confined within intergenic or un-annotated genomic regions. This poses a limit to the systematic functional classification of variants and a prompt identification of putative disease-causing mutations. The equivalent figure for an exome is in the order of about 80,000 variants per individual. Importantly, all these variants fall by definition within or close to functional genomic regions, and their effects can be predicted on the base of existing genomic annotations. The significance of a large number of intergenic variants detected by WGS remains unclear in large-scale clinical set-ups, as more than 80% of currently known disease-causing mutations are found within protein coding genes. This figure could be, however, an over-estimate due to ascertainment bias, since the majority of large-scale human genome resequencing projects aimed at the detection of disease-causing mutations have been carried out by means of WES sequencing, and a significant proportion of the studies was focused on rare monogenic Mendelian diseases. In this respect data, produced by WGS offer a more granular representation of the genomic variability, facilitating a more accurate reconstruction of the haplotypes which can be instrumental for the detection of genomic loci associated with complex phenotypic traits, including diseases like atherosclerosis, diabetes, and hypertension. Population genetics studies can benefit greatly from the wealth of genetic markers recovered from WGS sequencing, resulting in a more precise reconstruction of the evolutionary history of closely related populations.

Finally, another important advantage of WGS strategies is that they are not limited by any particular genomic annotation, and as such will probably form a better legacy for future investigations including newly discovered functional genomic elements. Indeed, although the current annotation of the human genome can be considered to be of high-quality, such a possibility can not be excluded as demonstrated by the recent explosion of the number of long non-coding RNA genes (Chen et al., 2016).

## Comparative Evaluation of Read Pre-Processing Strategies

Since the application of high-throughput sequencing technologies for the study of human genome variability at population scale level is becoming more and more commonplace, the development of standardized bioinformatics pipelines for an effective analysis the data is becoming crucial. Ideally, these pipelines should be fast, in order to cope with the increasing volumes of data, yet at the same time highly accurate as required by clinical applications. While implications of the usage of different combinations of tools for the alignment of short reads to the genome and for variant calling have been debated in depth (Pabinger et al., 2014), the evaluation of how quality assessment procedures can concur to the improvement of bioinformatic strategies for genotyping has been so far a little bit neglected. Indeed, good practices for quality assessment and pre-processing of the reads can contribute significantly to the optimization of downstream genotyping strategies, both by reducing computational requirements and by possibly lowering false positive rates. Three major approaches are commonly used for the pre-processing of reads obtained from large-scale resequencing studies: *quality trimming*, that is the polishing of the reads based on descriptive statistics calculated on their quality scores; *PCR de-duplication*, consisting in the elimination of identical reads or read pairs that might derive from PCR amplification of the same DNA fragment; *merging of overlapping pairs*, that consolidates pairs of reads originating from DNA fragments shorter than the combined length of the mates, into a longer, non-redundant sequence.

### Materials and Methods

In order to explore the impact of reads pre-processing strategies on genotyping workflows and devise guidelines and suggestions for its optimization, we took advantage of a collection of publicly available genome and exome (Nextera kit) sequencing data derived form the platinum genome NA12878 (Eberle et al., 2016). Reference call-sets along with genome and exome sequencing data were retrieved from the Illumina BaseSpace Sequence Hub^5^. Reads were preprocessed by using nine different pipelines (summarized in **Table 1**), based on the combination of three progressive quality trimming stringency levels, and by adopting or discarding PCR de-duplication and read merging steps. Computations were performed on a Centos linux server with 64 Gb of RAM and 24 CPU cores, using a limit of 12 CPU cores and 32 Gb of RAM for each step of the pipelines.

**Table 1:** Read pre-processing strategies used in this study.

Pre-processing strategy^∗^	Quality trimming^∗∗^	Merging of overlapped pairs^∗∗∗^	PCR de-duplication^∗∗∗∗^
Lax	Lead:Q20, Trail:Q15, Wlen:10,Q15	No	No
Medium	Lead:Q25, Trail:Q20, Wlen:15,Q20	No	No
Hard	Lead:Q25, Trail:Q25, Wlen:20,Q25	No	No
Lax + Ovl	Lead:Q20, Trail:Q15, Wlen:10,Q15	Min Ovl 15 bp	No
Medium + Ovl	Lead:Q25, Trail:Q20, Wlen:15,Q20	Min Ovl 15 bp	No
Hard + Ovl	Lead:Q25, Trail:Q25, Wlen:20,Q25	Min Ovl 15 bp	No
Lax + PCR	Lead:Q20, Trail:Q15, Wlen:10,Q15	No	MDR = 0.03
Medium + PCR	Lead:Q25, Trail:Q20, Wlen:15,Q20	No	MDR = 0.03
Hard + PCR	Lead:Q25, Trail:Q25, Wlen:20,Q25	No	MDR = 0.03

*From left to right, columns contain:*

*^∗^Description of the strategy, as described in main text and figures.*

*^∗∗^Trimmomatic parameters for quality trimming. Q, quality score cut-off; Wlen, length of the window for sliding window operations.*

*^∗∗∗^Pear parameters for read merging. Min Ovl, minimum overlap required for merging of read pairs.*

*^∗∗∗∗^MarkDuplicates parameters for PCR de-duplication. MDR (MAX DIFF RATE), overall mismatch rate for non-duplicated reads.*

Quality trimming was carried out using the Trimmomatic software (Bolger et al., 2014). Three different quality trimming procedures, with increasing levels of stringency, were used for the quality trimming of the raw reads. All the procedures were based on the same combination of Trimmomatic operations: “Leading” which removes nucleotides from the 5′ end of the reads if their quality score falls below a predefined cutoff, “Trailing” which performs the equivalent operations on the 3′ end of the reads, and “Slidingwindows,” which evaluates the average quality score of the reads along sliding windows of fixed length, cutting the read if the average quality score within a window falls below a given threshold. Reads resulting in less than 50 bps after quality trimming were not incorporated in the subsequent stages of the analyses. Different levels of stringency were implemented with the following parameters:

• Lax: Leading Qs >= 20; Trailing Qs > 15; Slidingwindows, windows length 10, Qs > 15.
• Medium: Leading Qs >= 25; Trailing Qs > 20; Slidingwindows, windows length 15, Qs > 20.
• Hard: Leading Qs >= 25; Trailing Qs > 25; Slidingwindows, windows length 20, Qs > 25.

Merging of overlapping paired end reads was performed with the PEAR (Zhang et al., 2014) program, using default parameters. Removal of potential PCR duplicates from exome sequencing data was performed with the MarkDuplicate module of the Picard software (Wysoker et al., 2013) with default parameters. Genotyping was performed by using the GATK workflow (DePristo et al., 2011), Varscan2 (Koboldt et al., 2012), and Freebayes (Garrison and Marth, 2012). Only variants supported by at least two methods were included in the final call-sets. Intersections and comparisons of call-sets were performed by means of the vcf-tools merge utility (Danecek et al., 2011) and bedtools intersect program (Quinlan and Hall, 2010). Reads were mapped to the reference hg38 human assembly using Bowtie2 (Langmead et al., 2009), and resulting bam files were preprocessed following the GATK best practices recommendations. Different levels of coverage (20–90x) were simulated by sub-sampling the reads. Pipelines were evaluated both in terms of computational requirements, accuracy and specificity of the results, by comparing the respective call-sets with the golden standard sets of variants provided by Illumina. For exome data, only variants falling within the target regions were considered.

### Impact of Reads Pre-processing Strategies on Variant Calling

**FIGURE 1:**
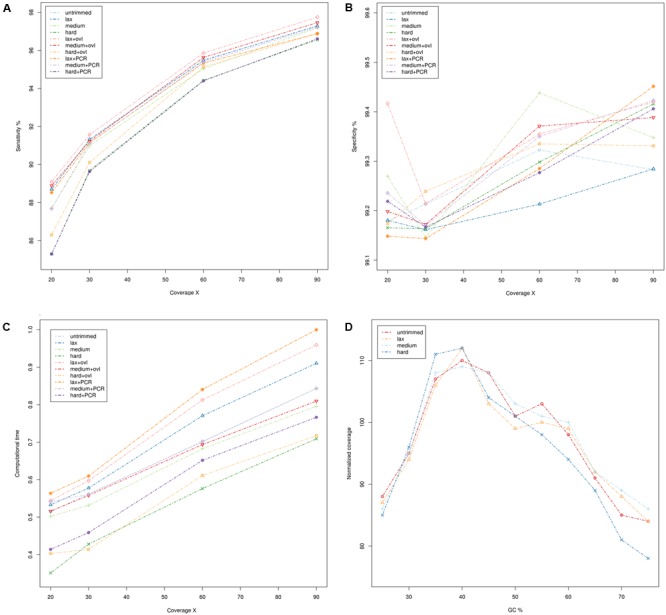
Comparison of quality assessment pipelines on Whole Genome Sequencing data. **(A)** Sensitivity of quality assessment pipelines at different levels of coverage. **(B)** Specificity of quality assessment pipelines at different levels of coverage. **(C)** Computation time required by the pipelines. Times reported have been scaled with unity-based normalization. **(D)** Normalized coverage levels with respect to GC composition. Coverage levels and GC composition were calculated on genomic windows of 200 bp, overlapping by 100 bp. Coverage levels were normalized using the upper-quartile normalization and scaled to 100 (where 100 represents the expected coverage).

**FIGURE 2:**
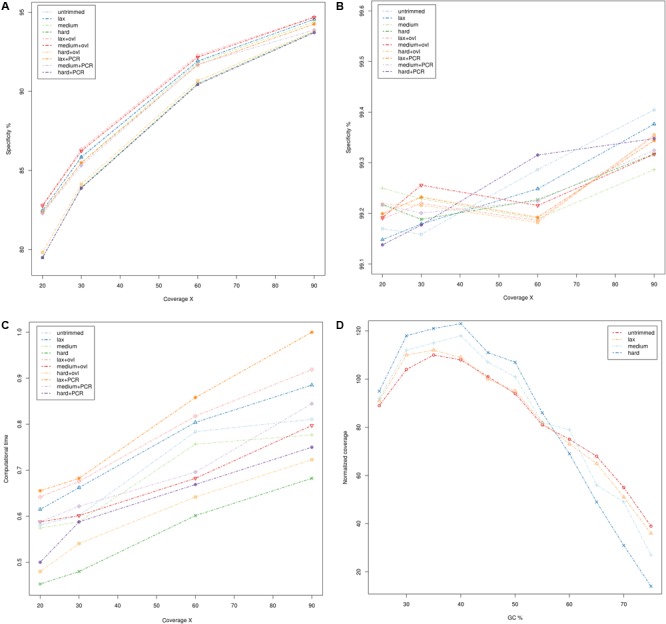
Comparison of quality assessment pipelines on Whole Exome Sequencing data. **(A)** Sensitivity of quality assessment pipelines at different levels of coverage. **(B)** Specificity of quality assessment pipelines at different levels of coverage. **(C)** Computation time required by the pipelines. Times reported have been scaled with unity-based normalization. **(D)** Normalized coverage levels with respect to GC composition. Coverage levels and GC composition were calculated on genomic windows of 200 bp, overlapping by 100 bp. Coverage levels were normalized using the upper-quartile normalization and scaled to 100 (where 100 represents the expected coverage).

The results are summarized in **Figure 1** (whole genome sequencing), **Figure 2** (WES) and detailed in Supplementary Tables S1–S5. Sensitivity and specificity of the call-sets obtained starting from the quality trimming strategies used in this study and described in the previous section are represented in **Figures 1A,B** for WGS and **Figures 2A,B** for WES datasets. Consistently with previous observations (Belkadi et al., 2015), WGS call-sets show a substantially higher sensitivity than WES, regardless of the (simulated) coverage level. This fact is probably due to a more uniform coverage profile (**Figures 1D, 2D**), where we can observe a considerable reduction in coverage of GC rich regions in the WES data. Interestingly, regions with a high GC content are affected by a systematic reduction in coverage across all the simulated depths of sequencing, and even at 90x we observe that only about half (45%) of the regions with a GC composition greater than 60% reach the minimal coverage of 20x required for the confident identification of heterozygous variants. Notably, quality trimming seems to cause a substantial reduction of GC rich regions, and in particular hard filters cause a pronounced decrease in coverage in the most GC rich genomic regions, on both WES and WGS data sets.

Per base quality score distributions did not indicate any significant differences between the overall quality profile of the calls corresponding to each nucleotide (not shown). However, we can notice a considerable reduction in the average quality scores of GC rich reads (content in GC >= 60%), suggesting that the drop in sequencing quality is restricted to specific sequence contexts. This is again consistent with previous observations, as the accuracy of Illumina sequencing technology is known to deteriorate slightly in the presence of GC rich sequences (Ross et al., 2013).

The specificity of the call-sets is between 99 and 99.5% and nearly identical across all coverage levels (**Figures 2A,B**), suggesting that the genotyping strategy used in this study is robust and can produce consistent results.

On the other hand, higher coverage levels are associated with a steady increase in sensitivity both for the WES and WGS data, suggesting that an adequate coverage is a key factor for the correct identification of genetic variants. Consistently with this observation, call-sets based on lax quality trimming, which resulted in a moderated reduction of the nominal coverage (**Figures 1A, 2A** and Supplementary Tables S4, S5), show a better sensitivity than equivalent sets where a more stringent quality trimming procedure was applied, and recover a higher proportion of “true” small indels and SNVs. More aggressive quality trimming can result in drastic reduction in coverage, with a systematic loss of accuracy in the most GC rich portions of the genome. This is particularly evident on the exome dataset, where the coverage is more skewed and influenced by GC composition. Interestingly, quality trimming resulted in a higher proportion of uniquely mapped reads, compared with the untrimmed data (Supplementary Tables S4, S5), suggesting that the removal of sequencing errors can improve the mappability of short reads.

Merging of overlapping reads pairs resulted in a small but general improvement of the sensitivity, leading to the identification of thousands of additional true variants (average 0.34% corresponding to 14,086 variants). In our experimental setup the application of this procedure yielded also a small (from 0.3 to 0.6%) but consistent increase in the number of reads mapping to the reference genome. Importantly, the majority (72.81%) of such reads were mapped to scarcely covered regions (average coverage 7.8x), which explains the increased sensitivity observed. Also, a significant proportion of the additional variants (52%) recovered by pipelines with overlapping read merging is represented by small indels, and occurs in highly variable genomic regions containing a complex combination of relatively short variants (28%). This suggests that the increased length of the merged reads can improve the alignment of such reads over highly polymorphic regions and facilitate the detection of complex events.

The usage of PCR de-duplication procedures seems to have, if any, only negative effects in all datasets analyzed in the present study: call-sets derived from pipelines where this step was applied show a marginal reduction in sensitivity at the cost of a general increase in computational times. Notably, we observe that even if all WGS resequencing data used in this study were produced by the means of a PCR free protocol, potential PCR duplicate reads are still identified even on these datasets, suggesting that the PCR de-duplication algorithm used in this study might be too stringent in the detection of potentially duplicated reads.

All the pipelines required comparable computational times (**Figures 1C, 2C** and Supplementary Tables S1, S2), and the additional computational overheads needed to perform the various pre-processing steps did not result in any relevant increase in computational resources. PCR de-duplication and merging of overlapping reads were the most demanding steps in this respect, yielding an average increase of the computational time by 28 and 31%, respectively. On the other hand, very stringent quality trimming resulted in a consistent reduction of computational requirements, however, at the price of a considerable drop in sensitivity.

Although we do not observe marked differences between the nine pipelines tested in this study, the pipeline based on permissive quality trimming of the reads coupled with merging of overlapping read pairs achieved a slightly improved sensitivity over the others, resulting in the identification of about 8,000 unique true variants that were otherwise missed. The pipeline based on medium stringency quality trimming and on merging of overlapping reads came as a close second, consistent with the idea that merging of overlapping reads can contribute to increase systematically the sensitivity of genotyping procedures, yet by a small margin.

## Conclusion

While population scale genome sequencing projects promise major revolutions in medical science, the need to efficiently analyze, handle and store such an unprecedented stream of data poses some major challenges which are still mostly unresolved. Indeed, bioinformatic analysis of NGS resequencing data is currently one of the major bottlenecks, limiting the development of swift and effective diagnostic tools based on of large-scale sequencing.

The analysis usually requires elaborate pipelines, where multiple tools need to be properly combined with the right parameters in order to obtain reliable results. Human genome resequencing data can be highly heterogeneous due to inherent biases introduced by different library preparation protocols, sequencing platforms and experimental strategies. As a consequence, the optimization of bioinformatic pipelines for the analysis of NGS resequencing data is highly desirable, as it can contribute to the reduction of the impact of systematic biases, and simultaneously maximize the outcome of the experiments. While several studies evaluated the performances of different variant calling pipelines, as of today we are not aware of dedicated studies performing a systematic evaluation of read quality pre-processing procedures. Good practices for quality assessment and pre-processing of the reads can contribute significantly to the optimization of genotyping strategies, both by reducing computational requirements and by lowering false positive rates. In this article we discussed advantages and limitations of state of the art genome resequencing techniques, and by taking advantage of publicly available data we devised a series of suggestions and good practices for the pre-processing of sequencing reads that can improve systematically the efficacy of bioinformatic genotyping strategies. These suggestions are of general applicability, have a minimal impact on computational resources, and therefore they can be instrumental for the future design of highly scalable and efficient genotyping systems.

By comparing the results achieved by nine different pre-processing pipelines on a golden standard reference genome for which more than 4 millions highly accurate SNVs are available (Eberle et al., 2016), we evaluated the effects of common quality assessment procedures on genotyping, both in terms of accuracy of the resulting variant call-sets and in terms of the required computational resources. Pipelines were evaluated by simulating various levels of coverage depth, from shallow to deep. Unsurprisingly, we observed that the sensitivity of the genotyping assays increased with the depth of sequencing levels, suggesting that an adequate coverage is the key for the identification of genetic variants.

The genotyping workflows tested in this study, which are based on a combination of three popular variant calling algorithms, achieved a steady level of accuracy under all the scenarios herein tested, allowing an unbiased comparison of their sensitivity. Notably, quality trimming procedures that were applied using different level of stringency did not have any major impact on the accuracy of the call-sets, suggesting that variant calling algorithms are generally robust to sequencing errors. However, we also observed that after quality trimming a higher proportion of reads could be mapped unambiguously on the reference genome, supporting the idea that the removal of sequencing errors can facilitate reads mapping. Highly stringent quality trimming filters, discarding a significant proportion of the reads (average 36% of the reads, 41% of the total amount of sequence), resulted in a substantial reduction in coverage and as a consequence in a permanent deterioration in sensitivity. GC rich regions, where the composition in GC exceeded 55–60%, were more affected by stringent quality trimming, resulting in a systematic loss of coverage. This trend was particularly evident in WES data, where compositional biases in the coverage profile with a reduced coverage of GC rich regions are commonly introduced by PCR amplification (Ross et al., 2013).

Removal of reads potentially deriving from PCR duplication artifacts did not have any significant impact on the results for the datasets analyzed in this study, yielding only a modest reduction in sensitivity (due to loss of coverage) at the cost of a consistent increase in computational resources. This is in accordance to previous reports showing that PCR-deduplication has little impact on the overall accuracy of genotyping assays (Ebbert et al., 2016). This is, however, also probably due to the high-quality of the sequencing libraries used in the course of studies of this kind (including the current), and to the presence of a limited number of duplicated reads. PCR de-duplication is an important quality assessment step, which can be used to assess systematically the overall quality of a sequencing experiment. For this reason we do not advise to remove PCR de-duplication from bioinformatic workflows for quality assessment of NGS reads. On the other hand, we noticed that for DNA libraries with low PCR duplication levels such process can be detrimental to variant identification. In such cases is probably better to avoid PCR de-duplication at all, and perform variant calling directly from non-de-duplicated bam files.

Datasets where merging of overlapping reads pairs was performed resulted in small but steady increased sensitivity level, facilitating the identification of genetic variants falling in highly polymorphic genomic regions. Longer reads produced by this process were preferentially mapped to genomic regions that were scarcely covered by shorter un-merged reads, resulting in an increase of coverage in highly heterogeneous regions of the genome. The increase in computational resources required by this procedure is on the other hand moderate, and is fully justified in the light of the improvements in sensitivity, yielding the discovery of thousands of otherwise missed “true” genetic variants. Interestingly, we noticed that stringent quality trimming filters, by shortening the reads, can lead to a considerable reduction in the number of pairs of overlapping reads that can be merged with confidence (lax trimming 10.2%; hard trimming 5.8%). This indicates that merging of overlapping reads should be preferentially performed before the application of quality filters. In such a scenario, a Smith-Waterman alignment between the 3′ ends (where sequencing errors are more frequent) and the 5′ ends of the R2 reads (which are generally of higher quality) can be used as an effective error correction procedure.

In conclusion, our experiments suggest that quality assessment procedures can have a considerable impact on the accuracy and sensitivity of human genome genotyping based on NGS sequencing. Variant calling algorithms are generally robust to sequencing error and a high level of accuracy can be achieved when the prediction of multiple tools are combined. Coverage levels seem to be the most important factor affecting the sensitivity of this type of genotyping assays. In the light of these considerations, quality assessment procedures based on relaxed quality trimming of the reads combined with merging of overlapping reads pairs seems ideal, as it can contribute a systematic improvement of the coverage of specific genomic regions, resulting in the identification of an increased number of true variants in highly polymorphic genomic contexts.

## Author Contributions

MC devised the study, performed bioinformatic analyses and wrote the manuscript. GP devised the study and wrote the manuscript.

## Conflict of Interest Statement

The authors declare that the research was conducted in the absence of any commercial or financial relationships that could be construed as a potential conflict of interest.

## Footnotes

1 http://www.whitehouse.gov/precision-medicine

2 http://omicsmaps.com/

3 https://www.illumina.com/systems/hiseq-x-sequencing-system.html

4 https://community.10xgenomics.com/t5/10x-Blog/A-basic-introduction-to-linked-reads/ba-p/95

5 https://blog.basespace.illumina.com/category/datasets/

## References

[B1] Aird D. Ross M. G. Chen W.-S. Danielsson M. Fennell T. Russ C. (2011). Analyzing and minimizing PCR amplification bias in Illumina sequencing libraries. *Genome Biol.* 12 R18. 10.1186/gb-2011-12-2-r18 PMC3188800 21338519

[B2] Alkuraya F. S. (2014). Genetics and genomic medicine in Saudi Arabia. *Mol. Genet. Genomic Med.* 2 369–378. 10.1002/mgg3.97 25333061 PMC4190871

[B3] Alyass A. Turcotte M. Meyre D. (2015). From big data analysis to personalized medicine for all: challenges and opportunities. *BMC Med. Genomics* 8 33 10.1186/s12920-015-0108-y PMC4482045 26112054

[B4] Ammar R. Paton T. A. Torti D. Shlien A. Bader G. D. (2015). Long read nanopore sequencing for detection of HLA and CYP2D6 variants and haplotypes. *F1000Research.* 4 17 10.12688/f1000research.6037.1 PMC4392832 25901276

[B5] Au C. H. Wa A. Ho D. N. Chan T. L. Ma E. S. (2016). Clinical evaluation of panel testing by next-generation sequencing (NGS) for gene mutations in myeloid neoplasms. *Diagn. Pathol.* 11 11 10.1186/s13000-016-0456-8 PMC4722624 26796102

[B6] Bao R. Huang L. Andrade J. Tan W. Kibbe W. A. Jiang H. (2014). Review of current methods, applications, and data management for the bioinformatics analysis of whole exome sequencing. *Cancer Inform.* 13(Suppl. 2) 67–82. 10.4137/CIN.S13779 PMC4179624 25288881

[B7] Belkadi A. Bolze A. Itan Y. Cobat A. Vincent Q. B. Antipenko A. (2015). Whole-genome sequencing is more powerful than whole-exome sequencing for detecting exome variants. *Proc.* *Natl. Acad. Sci. U.S.A.* 28 5473–5478. 10.1073/pnas.1418631112 PMC4418901 25827230

[B8] Bentley D. R. Balasubramanian S. Swerdlow H. P. Smith G. P. Milton J. Brown C. G. (2008). Accurate whole human genome sequencing using reversible terminator chemistry. *Nature* 456 53–59. 10.1038/nature075171038/nature07517 18987734 PMC2581791

[B9] Bolger A. M. Lohse M. Usadel B. (2014). Trimmomatic: a flexible trimmer for Illumina sequence data. *Bioinformatics* 30 2114–2120. 10.1093/bioinformatics/btu170 24695404 PMC4103590

[B10] Chaisson M. J. P. Huddleston J. Dennis M. Y. Sudmant P. H. Malig M. Hormozdiari F. (2015). Resolving the complexity of the human genome using single-molecule sequencing. *Nature* 517 608–611. 10.1038/nature13907 25383537 PMC4317254

[B11] Chen X. Yan C. C. Zhang X. You Z. H. (2016). Long non-coding RNAs and complex diseases:from experimental results to computational models. *Brief Bioinform.* 10.1093/bib/bbw060 [Epub ahead of print]. PMC5862301 27345524

[B12] Chilamakuri C. S. R. Lorenz S. Madoui M.-A. Vodák D. Sun J. Hovig E. (2014). Performance comparison of four exome capture systems for deep sequencing. *BMC Genomics* 15:449 10.1186/1471-2164-15-449 PMC4092227 24912484

[B13] Clark M. J. Chen R. Lam H. Y. K. Karczewski K. J. Chen R. Euskirchen G. (2011). Performance comparison of exome DNA sequencing technologies. *Nat. Biotechnol.* 29 908–914. 10.1038/nbt.1975 21947028 PMC4127531

[B14] Clarke J. Wu H. C. Jayasinghe L. Patel A. Reid S. Bayley H. (2009). Continuous base identification for single-molecule nanopore DNA sequencing. *Nat. Nanotechnol.* 4 265–270. 10.1038/nnano.2009.12 19350039

[B15] Collins R. L. Brand H. Redin C. E. Hanscom C. Antolik C. Stone M. R. (2017). Defining the diverse spectrum of inversions, complex structural variation, and chromothripsis in the morbid human genome. *Genome Biol.* 18 36 10.1186/s13059-017-1158-6 PMC5338099 28260531

[B16] Cornish A. Guda C. (2015). A comparison of variant calling pipelines using genome in a bottle as a reference. *BioMed Res. Int.* 2015 11 10.1155/2015/456479 PMC4619817 26539496

[B17] Cyranoski D. (2016). China embraces precision medicine on a massive scale. *Nature* 7 9–10. 10.1038/529009a 26738574

[B18] Danecek P. Auton A. Abecasis G. Albers C. A. Banks E. DePristo M. A. (2011). The variant call format and VCFtools. *Bioinformatics* 27 2156–2158. 10.1093/bioinformatics/btr330 21653522 PMC3137218

[B19] De Koning A. P. J. Gu W. Castoe T. A. Batzer M. A. Pollock D. D. (2011). Repetitive elements may comprise over two-thirds of the human genome. *PLoS Genet.* 7:e1002384 10.1371/journal.pgen.1002384 PMC3228813 22144907

[B20] De Leng W. W. J. Gadellaa-van Hooijdonk C. G. Barendregt-Smouter F. A. S. Koudijs M. J. Nijman I. Hinrichs J. W. (2016). Targeted next generation sequencing as a reliable diagnostic assay for the detection of somatic mutations in tumours using minimal DNA amounts from formalin fixed paraffin embedded material. *PLoS ONE* 11:e0149405 10.1371/journal.pone.0149405 PMC4769293 26919633

[B21] DePristo M. Banks E. Poplin R. Garimella K. V. Maguire J. R. Hartl C. (2011). A framework for variation discovery and genotyping using next-generation DNA sequencing data. *Nat. Genet.* 43 491–498. 10.1038/ng.8061038/ng.806 21478889 PMC3083463

[B22] Ebbert M. T. W. Wadsworth M. E. Staley L. A. Hoyt K. L. Pickett B. Miller J. (2016). Evaluating the necessity of PCR duplicate removal from next-generation sequencing data and a comparison of approaches. *BMC Bioinformatics* 17(Suppl. 7) 239 10.1186/s12859-016-1097-31186/s12859-016-1097-3 PMC4965708 27454357

[B23] Eberle M. A. Fritzilas E. Krusche P. Källberg M. Moore B. L. Bekritsky M. A. (2016). A reference data set of 5.4 million human variants validated by genetic inheritance from sequencing a three-generation 17-member pedigree. *Genome Res.* 27 157–164. 10.1101/gr.210500.116 27903644 PMC5204340

[B24] Eid J. Fehr A. Gray J. Luong K. Lyle J. Otto G. (2009). Real-time DNA sequencing from single polymerase molecules. *Science* 2 133–138. 10.1126/science.1162986 19023044

[B25] García-García G. Baux D. Faugère V. Moclyn M. Koenig M. Claustres M. (2016). Assessment of the latest NGS enrichment capture methods in clinical context. *Sci. Rep.* 6:20948 10.1038/srep20948 PMC4750071 26864517

[B26] Garrison E. Marth G. (2012). Haplotype-based variant detection from short-read sequencing. arXiv:1207.3907

[B27] Gerlinger M. Rowan A. J. Horswell S. Larkin J. Endesfelder D. Gronroos E. (2012). Intratumor heterogeneity and branched evolution revealed by multiregion sequencing. *N. Engl. J. Med.* 366 883–892. 10.1056/NEJMoa1113205 22397650 PMC4878653

[B28] Goodwin S. McPherson J. D. McCombie W. R. (2016). Coming of age: ten years of next-generation sequencing technologies. *Nat. Rev. Genet.* 17 333–351. 10.1038/nrg.2016.49 27184599 PMC10373632

[B29] Guo Y. Samuels D. C. Li J. Clark T. Li C. I. Shyr Y. (2013). Evaluation of allele frequency estimation using pooled sequencing data simulation. *Sci. World J.* 2013 895496 10.1155/2013/895496 PMC3582166 23476151

[B30] Gurdasani D. Carstensen T. Tekola-Ayele F. Pagani L. Tachmazidou I. Hatzikotoulas K. (2015). The african genome variation project shapes medical genetics in Africa. *Nature* 517 327–332. 10.1038/nature13997 25470054 PMC4297536

[B31] Huddleston J. Chaisson M. J. Meltz Steinberg K. Warren W. Hoekzema K. Gordon D. (2016). Discovery and genotyping of structural variation from long-read haploid genome sequence data. *Genome Res.* 27 677–685. 10.1101/gr.214007.116 27895111 PMC5411763

[B32] Hwang S. Kim E. Lee I. Marcotte E. M. (2015). Systematic comparison of variant calling pipelines using gold standard personal exome variants. *Sci. Rep.* 5:17875 10.1038/srep17875 PMC4671096 26639839

[B33] Katsanis S. H. Katsanis N. (2013). Molecular genetic testing and the future of clinical genomics. *Nat.* *Rev. Genet.* 14 415–426. 10.1038/nrg3493 PMC4461364 23681062

[B34] Kim S. T. Lee W. S. Lanman R. B. Mortimer S. Zill O. A. Kim K. M. (2015). Prospective blinded study of somatic mutation detection in cell-free DNA utilizing a targeted 54-gene next generation sequencing panel in metastatic solid tumor patients. *Oncotarget* 6 40360–40369. 10.18632/oncotarget.5465 26452027 PMC4741900

[B35] Koboldt D. C. Zhang Q. Larson D. E. Shen D. McLellan M. D. Lin L. (2012). VarScan 2: Somatic mutation and copy number alteration discovery in cancer by exome sequencing. *Genome Res.* 22 568–576. 10.1101/gr.129684.1111101/gr.129684.111 22300766 PMC3290792

[B36] Kotze M. J. Lückhoff H. K. Peeters A. V. Baatjes K. Schoeman M. van der Merwe L. (2015). Genomic medicine and risk prediction across the disease spectrum. *Crit. Rev. Clin. Lab. Sci.* 52 120–137. 10.3109/10408363.2014.997930 25597499

[B37] Langmead B. Trapnell C. Pop M. Salzberg S. L. (2009). Ultrafast and memory-efficient alignment of short DNA sequences to the human genome. *Genome Biol.* 10:R25 10.1186/gb-2009-10-3-r25 PMC2690996 19261174

[B38] Lek M. Karczewski K. J. Minikel E. V. Samocha K. E. Banks E. Fennell T. (2016). Analysis of protein-coding genetic variation in 60,706 humans. *Nature* 18 285–291. 10.1038/nature19057 PMC5018207 27535533

[B39] Lu Y. F. Goldstein D. B. Angrist M. Cavalleri G. (2014). Personalized medicine and human genetic diversity. *Cold Spring Harb. Perspect. Med.* 4 a008581 10.1101/cshperspect.a008581 PMC4143101 25059740

[B40] McCoy R. C. Taylor R. W. Blauwkamp T. A. Kelley J. L. Kertesz M. Pushkarev D. (2014). Illumina TruSeq synthetic long-reads empower de novo assembly and resolve complex, highly-repetitive transposable elements. *PLoS ONE* 9:e106689 10.1371/journal.pone.0106689 PMC4154752 25188499

[B41] Meienberg J. Bruggmann R. Oexle K. Matyas G. (2016). Clinical sequencing: is WGS the better WES? *Hum. Genet.* 135 359–362. 10.1007/s00439-015-1631-9 26742503 PMC4757617

[B42] Mostovoy Y. Levy-Sakin M. Lam J. Lam E. T. Hastie A. R. Marks P. (2016). A hybrid approach for de novo human genome sequence assembly and phasing. *Nat.* *Methods* 13 587–590. 10.1038/nmeth.3865 PMC4927370 27159086

[B43] Nagasaki M. Yasuda J. Katsuoka F. Nariai N. Kojima K. Kawai Y. (2015). Rare variant discovery by deep whole-genome sequencing of 1,070 japanese individuals. *Nat. Commun.* 6 8018 10.1038/ncomms90181038/ncomms9018 PMC4560751 26292667

[B44] Ng S. B. Turner E. H. Robertson P. D. Flygare S. D. Bigham A. W. Lee C. (2009). Targeted capture and massively parallel sequencing of twelve human exomes. *Nature* 461 272–276. 10.1038/nature08250 19684571 PMC2844771

[B45] Pabinger S. Dander A. Fischer M. Snajder R. Sperk M. Efremova M. (2014). A survey of tools for variant analysis of next-generation genome sequencing data. *Brief. Bioinform.* 15 256–278. 10.1093/bib/bbs0861093/bib/bbs086 23341494 PMC3956068

[B46] Quinlan A. R. Hall I. M. (2010). BEDTools: a flexible suite of utilities for comparing genomic features. *Bioinformatics* 26 841–842. 10.1093/bioinformatics/btq033 20110278 PMC2832824

[B47] Richards S. Aziz N. Bale S. Bick D. Das S. Gastier-Foster J. (2015). Standards and guidelines for the interpretation of sequence variants: a joint consensus recommendation of the american college of medical genetics and genomics and the association for molecular pathology. *Genet. Med.* 17 405–424. 10.1038/gim.2015.30 25741868 PMC4544753

[B48] Ross M. G. Russ C. Costello M. Hollinger A. Lennon N. J. Hegarty R. (2013). Characterizing and measuring bias in sequence data. *Genome Biol.* 14:R51 10.1186/gb-2013-14-5-r51 PMC4053816 23718773

[B49] Samorodnitsky E. Jewell B. M. Hagopian R. Miya J. Wing M. R. Lyon E. (2015). Evaluation of hybridization capture versus amplicon-based methods for whole-exome sequencing. *Hum. Mutat.* 36 903–914. 10.1002/humu.228251002/humu.22825 26110913 PMC4832303

[B50] Samuels D. C. Han L. Li J. Quanghu S. Clark T. A. Shyr Y. (2013). Finding the lost treasures in exome sequencing data. *Trends Genet.* 29 593–599. 10.1016/j.tig.2013.07.006 23972387 PMC3926691

[B51] Saudi Mendeliome Group (2015). Comprehensive gene panels provide advantages over clinical exome sequencing for Mendelian diseases. *Genome Biol.* 16 134 10.1186/s13059-015-0693-2 PMC4499193 26112015

[B52] Schirmer M. Ijaz U. Z. D’Amore R. Hall N. Sloan W. T. Quince C. (2015). Insight into biases and sequencing errors for amplicon sequencing with the Illumina MiSeq platform. *Nucleic Acids Res.* 43 e37 10.1093/nar/gku1341 PMC4381044 25586220

[B53] Sherry S. T. Ward M. H. Kholodov M. Baker J. Phan L. Smigielski E. M. (2001). dbSNP: the NCBI database of genetic variation. *Nucleic Acids Res.* 29 308–311. 11125122 10.1093/nar/29.1.308 PMC29783

[B54] Shigemizu D. Momozawa Y. Abe T. Morizono T. Boroevich K. A. Takata S. (2015). Performance comparison of four commercial human whole-exome capture platforms. *Sci. Rep.* 5:12742 10.1038/srep12742 PMC4522667 26235669

[B55] Sidore C. Busonero F. Maschio A. Porcu E. Naitza S. Zoledziewska M. (2015). Genome sequencing elucidates sardinian genetic architecture and augments association analyses for lipid and blood inflammatory markers. *Nat. Genet.* 47 1272–1281.111 10.1038/ng.3368 26366554 PMC4627508

[B56] Stephens Z. D. Lee S. Y. Faghri F. Campbell R. H. Zhai C. Efron M. J. (2015). Big data: astronomical or genomical? *PLoS Biol.* 13:e1002195 10.1371/journal.pbio.1002195 PMC4494865 26151137

[B57] Taub M. A. Corrada Bravo H. Irizarry R. A. (2010). Overcoming bias and systematic errors in next generation sequencing data. *Genome Med.* 2 87 10.1186/gm208 PMC3025429 21144010

[B58] The 1000 Genomes Project Consortium Auton A. Brooks L. D. Durbin R. M. Garrison E. P. Kang H. M. (2015). A global reference for human genetic variation. *Nature* 526 68–74. 10.1038/nature15393 26432245 PMC4750478

[B59] UK10K Consortium Walter K. Min J. L. Huang J. Crooks L. Memari Y. (2015). The UK10K project identifies rare variants in health and disease. *Nature* 526 82–90. 10.1038/nature14962 26367797 PMC4773891

[B60] Weisenfeld N. I. Kumar V. Shah P. Church D. M. Jaffe D. B. (2017). Direct determination of diploid genome sequences. *Genome Res.* 27 757–767. 10.1101/gr.214874.116 28381613 PMC5411770

[B61] Wysoker A. Tibbetts K. Fennell T. (2013). *Picard Tools Version 1.90.* Available at: http://picard.sourceforge.net

[B62] Zhang G. Wang J. Yang J. Li W. Deng Y. Li J. (2015). Comparison and evaluation of two exome capture kits and sequencing platforms for variant calling. *BMC Genomics* 16 581 10.1186/s12864-015-1796-6 PMC4524363 26242175

[B63] Zhang J. Kobert K. Flouri T. Stamatakis A. (2014). PEAR: a fast and accurate Illumina Paired-End reAd mergeR. *Bioinformatics* 30 614–620. 10.1093/bioinformatics/btt593 24142950 PMC3933873

